# QPX7728, An Ultra-Broad-Spectrum B-Lactamase Inhibitor for Intravenous and Oral Therapy: Overview of Biochemical and Microbiological Characteristics

**DOI:** 10.3389/fmicb.2021.697180

**Published:** 2021-07-05

**Authors:** Olga Lomovskaya, Ruslan Tsivkovski, Dongxu Sun, Raja Reddy, Maxim Totrov, Scott Hecker, David Griffith, Jeffery Loutit, Michael Dudley

**Affiliations:** ^1^Qpex Biopharma, Inc., San Diego, CA, United States; ^2^Molsoft, LLC, San Diego, CA, United States

**Keywords:** QPX7728, beta-lactamase inhibitor, carbapenemase, metallo β-lactamase, CRE, Acinetobacter, Pseudomonas

## Abstract

QPX7728 is a novel β-lactamase inhibitor (BLI) that belongs to a class of cyclic boronates. The first member of this class, vaborbactam, is a BLI in the recently approved Vabomere (meropenem-vaborbactam). In this paper we provide the overview of the biochemical, structural and microbiological studies that were recently conducted with QPX7728. We show that QPX7728 is an ultra-broad-spectrum β-lactamase inhibitor with the broadest spectrum of inhibition reported to date in a single BLI molecule; in addition to potent inhibition of clinically important serine β-lactamases, including Class A and D carbapenemases from Enterobacterales and notably, diverse Class D carbapenemases from Acinetobacter, it also inhibits many metallo β-lactamases. Importantly, it is minimally affected by general intrinsic resistance mechanisms such as efflux and porin mutations that impede entry of drugs into gram-negative bacteria. QPX7728 combinations with several intravenous (IV) β-lactam antibiotics shows broad coverage of Enterobacterales, *Acinetobacter baumannii* and *Pseudomonas aeruginosa*, including strains that are resistant to other IV β-lactam-BLI combinations, e.g., ceftazidime-avibactam, ceftolozane-tazobactam, meropenem-vaborbactam and imipenem-relebactam that were recently approved for clinical use. Based on studies with *P. aeruginosa*, different partner β-lactams in combination with QPX7728 may be optimal for the coverage of susceptible organisms. This provides microbiological justification for a stand-alone BLI product for co-administration with different β-lactams. QPX7728 can also be delivered orally; thus, its ultra-broad β-lactamase inhibition spectrum and other features could be also applied to oral QPX7728-based combination products. Clinical development of QPX7728 has been initiated.

## Introduction

Development of β-lactamase inhibitors (BLIs) in combination with β-lactams continues to be a powerful strategy to restore potency of β-lactams against β-lactamase-producing bacteria, thus extending clinical utility of this important class of antibiotics (Bush, 2018; Bush and Bradford, 2019; Ho et al., 2019; Papp-Wallace, 2019). Recent approval of four BL/BLI combinations represents significant progress in the successful development of new IV agents to address infections due to gram-negative pathogens (Talbot et al., 2019) identified by both the Centers for Disease Control (CDC) and the World Health Organization (WHO) as urgent or serious threats (CDC, 2019) for which new agents are critically needed (WHO, 2017). However, while new BLIs, avibactam, relebactam, and vaborbactam, have a much broader spectrum of β-lactamase inhibition compared to clavulanic acid, tazobactam and sulbactam, they have no activity against metallo β-lactamases; consequently, new BL/BLI combinations have no utility in treatment of infections due to MBL-producing strains of Enterobacterales unless they are co-administered with a monobactam like aztreonam (Bush and Bradford, 2019; Papp-Wallace, 2019). Of note, recent reports have demonstrated an increasing number of CRE producing the New Delhi metallo-β-lactamase (NDM-1), notably in COVID-19 patients (Nori et al., 2020; Porretta et al., 2020). In addition, none of the recently approved BLIs have activity against Class D carbapenemases from *Acinetobacter baumannii* and thus new BL/BLI combinations cannot be used to treat infections due to carbapenem-resistant Acinetobacter.

The increase in drug-resistant infections is not limited to hospital settings; multi-drug resistance to existing oral antibiotics (fluoroquinolones, trimethoprim-sulfamethoxazole, and β-lactams) is on the rise in community settings as well (Calzi et al., 2016; Mazzariol et al., 2017; Trautner, 2018; Critchley et al., 2019). Unfortunately, none of the BL/BLI combinations that recently became available for the IV treatment of gram-negative infections due to CRE and ESBL-producing Enterobacterales are orally bioavailable (Talbot et al., 2019).

Prompted by this ongoing unmet clinical need and encouraged by our experience with cyclic boronate class that gave rise to the first FDA-approved BLI vaborbactam from this class, we continued to optimize the pharmacophore associated with inhibition of β-lactamases. The main objective was to expand the spectrum of β-lactamase inhibition of cyclic boronic acid-based BLIs to include both serine and metallo β-lactamases. In addition, we explored structures that could achieve oral bioavailability for use in combination with oral β-lactams. Although two different molecules were originally envisioned, the SAR converged with the discovery of QPX7728 which allowed us to achieve both target profiles in a single molecule.

## The Discovery Path From Vaborbactam to Ultra-Broad-Spectrum QPX7728 Was a Combination of Rational Strategies and Unexpected Breakthroughs

**FIGURE 1:**
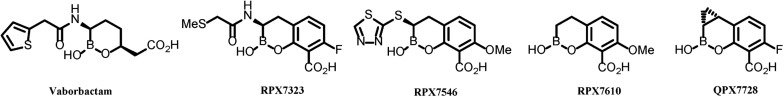
Evolution of boronic β-lactamase inhibitors.

Vaborbactam, the first boronic BLI approved as a combination product with meropenem, was specifically optimized for inhibition of KPC (Hecker et al., 2015). Its potency is ca. 10–50-fold less against other class A or class C beta lactamases compared to KPC enzymes (Tsivkovski and Lomovskaya, 2019). It has no activity against Class D or Class B β-lactamases. Soon after vaborbactam was introduced into clinical development, our discovery team began to work on the next generation of boronic BLIs starting with bicyclic amide series of compounds. This effort is described in detail by Hecker et al. (2020). Earlier studies demonstrated that related compounds possessed broad-spectrum inhibition of serine β-lactamases (Ness et al., 2000) and our own modeling indicated that broadening of the spectrum to include metallo enzymes (MBLs) was possible. Indeed, the amide series represented by RPX7323 (Figure 1) were capable of potently inhibiting serine enzymes from classes A-D and there were analogs that afforded weak activity against metallo enzymes (MBLs, Class B).

The first real breakthrough came from a completely opportunistic discovery of bicyclic thioether series of compounds, as exemplified by RPX7546 (Figure 1), in which the amide linkage is replaced by a thio linkage (Reddy et al., 2014, 2016). The very first sulfur-linked compound in this series was prepared primarily because it was easy to synthesize. This replacement unexpectedly achieved improved potency against MBLs. Further optimization centered around gaining activity against Class D carbapenemases from Acinetobacter (*e.g.*, OXA-23/OXA-24/OXA-58).

The next breakthrough came with the discovery of RPX7610 (Figure 1), a compound that lacked a substituent next to boron: it achieved potent inhibition of all target β-lactamases and also appeared to be minimally affected by efflux and porin mutations. Ironically, this unsubstituted analog was prepared as a model compound for BLIs with a heteroatom in the aryl ring; its potency and spectrum were unexpected. Advanced evaluation of this compound identified, however, its metabolic liability. This called for a rational approach to identify moieties that would confer metabolic stability but would not be detrimental for the achieved advantageous characteristics of RPX7610. This effort culminated in the discovery of QPX7728 (Hecker et al., 2020).

### QPX7728 With High Potency Inhibits Multiple Serine and Metallo-β-Lactamases

The potency of inhibition of several purified β-lactamases from structural Classes A, C and D and MBLs from Class B by QPX7728 was studied by Tsivkovski et al. (2020) (Table 1). IC_50_ values were in low nM range for class A KPC-2, CTX-M-15, SHV-12, TEM-43 and in double digit nM range for Class C P99. QPX7728 also inhibited with high potency (IC_50_ in low nM range) all tested Class D carbapenemases, such as OXA-48 from Enterobacterales and, importantly, OXA-23 from *A. baumannii*.

**TABLE 1:** Potency (IC_50_, nM) of β-lactamases inhibition by QPX7728 compared to other BLIs.

Enzyme	Class	CARB	Vaborbactam	Avibactam	Relebactam	QPX7728
KPC-2	A	+	110 ± 30	22 ± 6	82 ± 17	2.9 ± 0.4
CTX-M-14	A	−	110 ± 40	1.4 ± 0.4	34 ± 10	0.94 ± 0.2
CTX-M-15	A	−	92 ± 13	0.56 ± 0.25	76 ± 3	1.2 ± 0.1
SHV-12	A	−	56 ± 11	0.61 ± 0.19	330 ± 50	1.9 ± 0.6
TEM-10	A	−	470 ± 150	4.3 ± 1.2	160 ± 20	2.2 ± 0.8
P99	C	−	88 ± 38	26 ± 7	36 ± 4	22 ± 6
OXA-48	D	+	6.9 ± 2.3 × 10^3^	180 ± 50	9 ± 0.3 × 10^4^	1.1 ± 0.4
OXA-23	D	+	1.2 ± 0.2 × 10^5^	3.1 ± 0.6 × 10^3^	ND	1.2 ± 0.4
NDM-1*	B	+	>1.6 × 10^5^	>1.6 × 10^5^	>1.6 × 10^5^	55 ± 25
VIM-1	B	+	>1.6 × 10^5^	>1.6 × 10^5^	>1.6 × 10^5^	14 ± 4
IMP-1*	B	+	>1.6 × 10^5^	>1.6 × 10^5^	>1.6 × 10^5^	610 ± 70
IMP-26*	B	+	>1.6 × 10^5^	>1.6 × 10^5^	>1.6 × 10^5^	4.1 ± 1 × 10^3^

*Imipenem was used as a substrate. Data from Tsivkovski et al. (2020).

Notably, QPX7728 inhibited clinically important MBLs, VIM-1 and NDM-1 with double-digit nM potency. Somewhat lower inhibitory potency (IC_50_ in μM range) was observed for IMP MBLs (Table 1).

The strain of *P. aeruginosa* PAM1154 lacks the major efflux pump MexAB-OprM and was used by Lomovskaya et al. (2020b) as a host to construct a panel of over 55 recombinant strains producing plasmid encoded single β-lactamases. This panel confirmed the spectrum of β-lactamase inhibition by QPX7728 observed in biochemical assays and expanded the list of enzymes inhibited by QPX7728 (Table 2). For purposes of comparative characterization with different β-lactamase inhibitors, substrates such as ceftazidime and piperacillin were used due to their combined lability to β-lactamases from all classes.

**TABLE 2:** *In vitro* potency (MIC, μg/ml) of ceftazidime and piperacillin alone and in combination with BLIs against the panel of engineered strains producing single β-lactamases.

			Ceftazidime			Piperacillin		
Strain	B-lactamase	Class	none	w/avibactam	w/QPX7728	none	w/avibactam	w/QPX7728
PAM4175	pUCP24 vector	none	0.25	0.25	≤0.06	0.125	0.125	≤0.06
PAM4819	CTX-M-2	A	1	0.125	≤0.06	64	≤0.06	≤0.06
PAM4743	CTX-M-15	A	32	0.25	0.125	>64	0.125	≤0.06
PAM4820	CTX-M-25	A	8	0.25	0.125	>64	0.25	≤0.06
PAM4822	CTX-M-27	A	64	0.25	0.125	>64	0.25	≤0.06
PAM4886	GES-1	A	64	0.25	0.25	4	0.25	≤0.06
PAM4800	GES-19	A	>64	0.5	0.125	8	0.25	≤0.06
PAM4840	OXY-6-2	A	0.5	0.25	0.25	>64	2	≤0.06
PAM4842	PER-2	A	>64	0.5	0.125	1	0.125	≤0.06
PAM4907	PER-4	A	>64	>64	0.25	0.5	0.25	≤0.06
PAM4874	SHV-12	A	>64	0.25	0.25	>64	0.5	≤0.06
PAM4878	TEM-10	A	>64	0.25	0.125	32	0.125	≤0.06
PAM4908	VEB-1	A	>64	1	0.25	8	0.25	≤0.06
PAM4910	VEB-2	A	>64	1	0.25	16	0.25	≤0.06
PAM4912	VEB-3	A	>64	1	0.25	8	0.25	≤0.06
PAM4938	VEB-9	A	256	2	≤0.25	16	0.13	≤0.06
PAM4135	KPC-2	A-CARB	16	0.25	0.125	>64	1	≤0.06
PAM4689	KPC-3	A-CARB	>64	0.5	0.125	>64	1	≤0.06
PAM4794	NMC-1	A-CARB	1	0.25	0.125	16	0.5	≤0.06
PAM4864	SFC-1	A-CARB	2	0.25	0.125	>64	0.25	≤0.06
PAM4744	SME-2	A-CARB	0.5	0.25	0.125	8	0.5	≤0.06
PAM4938	VCC-1	A-CARB	ND	ND	ND	16	≤0.06	≤0.06
PAM4676	BKC-1	A-CARB	3	0.25	0.125	32	0.5	≤0.06
PAM4186	CMY-2	C	16	0.25	0.125	8	≤0.06	≤0.06
PAM4825	MIR-1	C	16	0.25	0.125	8	0.125	≤0.06
PAM4745	P99	C	64	0.25	0.125	64	0.125	≤0.06
PAM4869	PDC-1	C	16	0.25	0.125	>64	0.5	≤0.06
PAM4884	ADC-181	C	8	0.5	0.25	1	0.125	≤0.06
PAM4827	OXA-1	D	0.125	0.125	≤0.06	4	≤0.06	≤0.06
PAM4792	OXA-2	D	8	0.25	0.125	64	0.5	≤0.06
PAM4846	OXA-9	D	0.25	0.25	0.125	8	0.125	≤0.06
PAM4790	OXA-10	D	0.25	0.25	0.125	0.125	0.125	≤0.06
PAM4217	OXA-48	D-CARB	0.25	0.25	0.125	16	0.125	≤0.06
PAM4875	OXA-23	D-CARB	0.25	0.25	0.125	16	4	≤0.06
PAM4876	OXA-72	D-CARB	0.25	0.25	0.125	16	4	≤0.06
PAM4877	OXA-58	D-CARB	0.25	0.25	0.125	4	1	≤0.06
PAM4179	NDM-1	B1	>64	2048	32 (8)*	64	64	0.25
PAM4917	NDM-7	B1	>64	2048	32 (4)*	32	32	0.25
PAM4795	VIM-1	B1	64	64	0.25	16	16	≤0.06
PAM4798	VIM-2	B1	16	16	0.125	16	16	≤0.06
PAM4881	VIM-7	B1	0.5	0.25	0.25	2	2	≤0.06
PAM4887	IMP-1	B1	>64	>64	2	1	0.5	≤0.06
PAM4888	IMP-4	B1	>64	>64	2	0.5	0.5	≤0.06
PAM4196	IMP-13	B1	64	64	2	0.5	0.5	≤0.06
PAM4198	IMP-15	B1	64	64	1	0.5	0.25	≤0.06
PAM4890	IMP-19	B1	>64	>64	4	0.25	0.25	≤0.06
PAM4889	IMP-26	B1	>64	>64	64	0.125	0.125	≤0.06
PAM4879	CcrA1	B1	4	4	0.125	0.25	0.125	≤0.06
PAM4883	GIM-1	B1	>64	64	0.5	>64	>64	2
PAM4885	SPM-1	B1	>64	>64	>64	32	32	8
PAM4880	L1	B3	64	64	64	32	32	8

*Genes encoding various β-lactamases were cloned in the plasmid pUCP24 and introduced in the strain of P. aeruginosa that lacks major efflux pumps (Lomovskaya et al., 2020b). Avibactam and QPX7728 tested at 4 μg/mL; CARB, carbapenemase. The number in bracket correspond to ceftazidime in combination with QPX7728 at 8 μg/mlData from Lomovskaya et al. (2020b).*

Based on results of both biochemical and microbiological assays, QPX7728 is a potent inhibitor of Class A ESBLs such as CTX-M, SHV, TEM, VEB, and PER, including some variants such as PER-4 that are less susceptible to inhibition with avibactam. It also inhibits Class A carbapenemases identified in various species of gram-negative bacteria such as KPC, SME, NMC-A, GES-20, VCC-1, BKC-1. Its spectrum of inhibition includes many Class C β-lactamases, both plasmidic, such as SMY and MIR from Enterobacterales, and chromosomal enzymes from Enterobacterales, *P. aeruginosa* and *A. baumannii*. QPX7728 appears to be equally potent in inhibiting diverse Class D β-lactamases, including carbapenemases such as OXA-48 from Enterobacterales and OXA-23/24/72/58 from *A. baumannii*.

Importantly, QPX7728 inhibits many metallo-β-lactamases from the B1 (Galleni et al., 2001) sub-class of the Class B family including NDM, VIM, CcrA1, IMP, and GIM but not SPM-1. The B1 sub-class represents the most common MBL enzymes; there are two other classes of MBLs, B2 and B3, consisting of less common β-lactamases. There are significant structural and mechanistic differences between B1 vs. B2 and particularly, B3 MBLs (Galleni et al., 2001). B2 and B3 MBLs such as L1 from *Stenotrophomonas maltophilia* are not or poorly inhibited by QPX7728.

### QPX7728 Inhibits Serine and Metallo Enzymes With a Significantly Different Kinetics

Detailed kinetic studies performed with QPX7728 and described in Tsivkovski et al. (2020) indicated that inhibition of all the tested serine enzymes by QPX7728 was consistent with slow tight-binding mechanism associated with progressive inactivation. QPX7728 acts by a two-step inhibition mechanism in which a non-covalent complex is formed first and subsequently proceeds to a covalent interaction. It is expected that the boron atom of QPX7728 forms covalent bond with the catalytic serine residue of the β-lactamase. Such covalent complexes were noted for other boronic BLIs (Rojas et al., 2016). Based on kinetic analysis, QPX7728 is a highly efficient inhibitor of serine β-lactamases with K_*on*_s in a 10^4^ M^–1^ s^–1^ × 10^6^ M^–1^ s^–1^ range (Table 3). In the case of KPC-2 inhibition, QPX7728 appears to be almost 100-fold more efficient than vaborbactam [k_2_/K of 5.5 × 10^3^ (Tsivkovski and Lomovskaya, 2019)] and ca. 10-fold more efficient than avibactam [k_2_/K of 1.3 × 10^4^ (Ehmann et al., 2013)] and relebactam [k_2_/K of 2.5 × 10^4^ (Papp-Wallace et al., 2018)].

**TABLE 3:** Kinetic parameters of β-lactamase inhibition by QPX7728.

Enzyme	k_2_/K (M^–1^ s^–1^)	k_*off*_, s^–1^	Residence time, min	K_*d*_, nM	Stoichiometry
KPC-2	3.6 ± 0.1 × 10^5^	9.0 ± 1.4 × 10^–5^	189 ± 31	0.25 ± 0.03	1
KPC-3	4.1 ± 1.0 × 10^5^	1.26 ± 0.07 × 10**^–^**^4^	133 ± 8	0.31 ± 0.06	1
BKC-1	1.82 ± 0.04 × 10^6^	1.1 ± 0.1 × 10**^–^**^4^	154 ± 14	0.060 ± 0.005	1
FRI-1	1.15 ± 0.03 × 10^6^	1.2 ± 0.2 × 10**^–^**^4^	138 ± 24	0.11 ± 0.01	1
SME-2	1.2 ± 0.1 × 10^6^	1.8 ± 0.2 × 10**^–^**^4^	94 ± 11	0.15 ± 0.02	1
CTX-M-15	6.9 ± 0.6 × 10^5^	8.0 ± 1.0 × 10**^–^**^5^	220 ± 33	0.11 ± 0.02	1
SHV-12	1.1 ± 0.2 × 10^5^	3.0 ± 0.2 × 10**^–^**^3^	5.5 ± 0.3	28 ± 4	1
TEM-43	1.9 ± 0.3 × 10^6^	3.2 ± 0.2 × 10**^–^**^4^	53 ± 3	0.17 ± 0.02	1
P99	6.3 ± 0.7 × 10^4^	3.3 ± 0.3 × 10**^–^**^5^	506 ± 51	0.53 ± 0.06	1
OXA-48	2.75 ± 0.09 × 10^6^	3.6 ± 0.2 × 10**^–^**^4^	47 ± 3	0.13 ± 0.01	1
OXA-23	9.9 ± 0.6 × 10^5^	1.6 ± 0.2 × 10**^–^**^3^	11 ± 2	1.6 ± 0.2	2
OXA-24	1.5 ± 0.2 × 10^6^	9.0 ± 1.0 × 10**^–^**^4^	20 ± 3	0.58 ± 0.10	1
OXA-58	1.07 ± 0.08 × 10^6^	3.5 ± 0.3 × 10**^–^**^4^	4.8 ± 0.5	3.2 ± 0.3	1
NDM-1	ND	ND	ND	32 ± 14	ND
VIM-1	ND	ND	ND	7.5 ± 2.1	ND
IMP-1	ND	ND	ND	240 ± 30	ND

*ND, not determined. Data from Tsivkovski et al. (2020).*

QPX7728 inhibition of serine β-lactamases was found to be reversible in all the studied enzymes; however, the stability of the QPX7728-β-lactamase complexes differed depending on the enzyme. The longest target residence time, ∼3.5 h, was detected for CTX-M-15 and the shortest, 5–20 min, for OXA carbapenemases from *A. baumannii* (Table 3). The residence time of QPX7728 for KPC-2, 3 h, was slightly longer than that of avibactam (∼2 h) (Ehmann et al., 2013), and relebactam (1.5 h) (Papp-Wallace et al., 2018) but shorter than that for vaborbactam (∼6 h) (Tsivkovski and Lomovskaya, 2019). Despite the 2–3-fold lower *k*_*off*_ values of vaborbactam inhibition of KPC-2 and KPC-3, QPX7728 K_*d*_ values for these enzymes were 10 to 50-fold lower than vaborbactam owing to its ca. 100-fold higher inactivation efficiency of KPCs (Tsivkovski and Lomovskaya, 2019; Table 3).

While QPX7728 behaves as a two-step covalent slow off-rate inhibitor of serine enzymes, its inhibition of metallo can be described by a classical Michaelis-Menten kinetics with a simple one-step complex formation followed by rapid dissociation of the enzyme-inhibitor complex. It is a competitive inhibitor with no sign of progressive inactivation and with K_*i*_s ranging from ∼7 nM, ∼32 nM and ∼240 nM for VIM-1, NDM-1 and IMP-1, respectively (Table 3).

### Structural Studies Are Consistent With the Mechanism of QPX7728 Inhibition and Provide Insights Into Its Potency, Breadth of Spectrum, and Its Limitations

**FIGURE 2:**
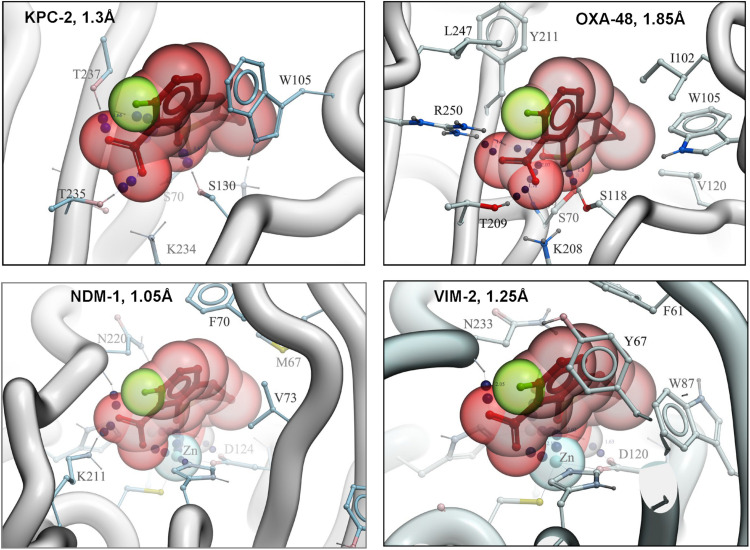
High resolution structures of QPX7728 with KPC-2, OXA-48, NDM-1, and VIM-2. Accession codes are the following: 6V1J (KPC-2: QPX7728), 6V1M (NDM-1: QPX7728), 6V1O (OXA-48: QPX7728), and 6V1P (VIM-2: QPX7728). Modified from Hecker et al. (2020).

The structures of KPC-2, NDM-1, VIM-2 and OXA-48 complexed with QPX7728 were solved at high resolution ranging from 1.05 to 1.85Å (Hecker et al., 2020). Structures of QPX7728 complexed to KPC-2, and OXA-48 provide clear evidence of the covalent bond between the boron atom of the inhibitor and the catalytic serine residue of the enzymes. In the metallo enzymes, the covalent bond is observed between the boron atom and the catalytic water molecule present in the active site of NDM-1 and VIM-2. Of note, the boron becomes tetrahedral on binding to both MBLs and SBLs (Figure 2).

**FIGURE 3:**
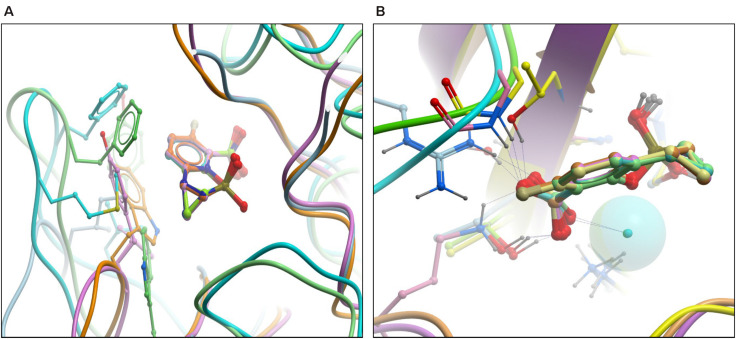
High resolution structures of RPX7728 with multiple enzymes: superimposition of β-lactamases using the ligand. **(A)** The aromatic/lipophilic interactions are shown. **(B)** Close-up of carboxylate interactions and orientation.

Superimposition of β-lactamases using the ligand shows that QPX7728 binds all of them in a nearly identical conformation. The most significant difference appears to be in the orientation of carboxylate in metallo- vs. non metallo enzymes; it is closer to co-planar in MBLs and closer to orthogonal in others (Figure 3). All enzymes engage the carboxylate in multiple strong interactions, but details can vary and there may be hydrogen bonds to side chain (Thr)hydroxyl, backbone amide or a water molecule as well as interactions with cationic side chains (Lys or Arg) or metal ion (Zn) (Figure 3B). Common to all β-lactamase enzymes are lipophilic interactions of the ‘back’ edge of the ring system, which is always packed against lipophilic, predominantly aromatic, sidechains which otherwise vary significantly from enzyme to enzyme (Figure 3A).

**FIGURE 4:**
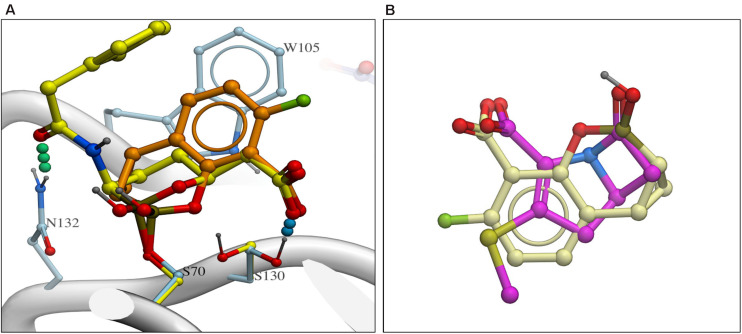
**(A)** Comparison of QPX7728 and vaborbactam in complex with KPC-2 with carbon atoms of QPX7728 and vaborbactam in light brown and in yellow carbon atoms, respectively [accession codes are the following: 6V1J (KPC-2:QPX7728); 6TD0 (KPC-2:vaborbactam)]. **(B)** Superposition of QPX7728 (crème carbon atoms) and the core ring system of penem antibiotics (magenta carbon atoms).

Recently the structure of the KPC-vaborbactam complex was published by two groups (Pemberton et al., 2020; Tooke et al., 2020) and there are both similarities and differences with KPC-QPX7728 complex (Figure 4A). Both boronates are covalently attached to S70 and the positioning of carboxylate is nearly identical but the boronate moieties are positioned somewhat differently. Vaborbactam inserts both exocyclic and endocyclic oxygen atoms into oxyanion hole, while only exocylic oxygen of QPX7728 is in the oxyanion hole. Endocyclic oxygen is coordinated by the S130 side chain. Overall QPX7728/KPC interactions are focused on the core conserved active site which QPX7728 fully occupies via the double ring system, forming more extensive lipophilic interactions with aromatic W105 side chain and other lipophilic surfaces. In contrast, vaborbactam picks up more interactions on the periphery, including a hydrogen bond to N132 and lipophilic contacts of the thiophene ‘sidechain.’

As discussed in Tsivkovski et al. (2020), a notable feature of QPX7728 molecular structure is its limited flexibility – the system of three rings constrains almost the entire molecule, and the only remaining free torsion (carboxylate rotation) is restricted by conjugation. Near-total rigidity of QPX7728 should result in a negligible conformational entropy loss upon binding, and likely contributes to its enhanced potency as compared to vaborbactam, which has 5 rotatable bonds. As evidenced by X-ray structures (Hecker et al., 2015), the vaborbactam ring also can adopt two alternative low energy conformations, for a total of 6 degrees of freedom. Assuming entropic loss of TΔS ∼0.6 kcal/mol per degree of freedom, the vaborbactam molecule might experience a penalty in binding free energy ΔG of as much as 3.6 kcal/mol, corresponding to a several hundred-fold difference in K_*d*_.

As suggested in the same study (Tsivkovski et al., 2020), one factor that likely contributes to the exceptional breadth of inhibition spectrum of QPX7728 is that its structure is very compact. The entire inhibitor molecule is only slightly larger than the β-lactam substrate core 5/4 ring system, and it fits within the well-conserved immediate vicinity of the catalytic site. Lack of any peripheral moieties in contact with more distal (and variable) regions of the enzyme may allow the inhibitor to avoid unfavorable interactions that sequence/structure variations could otherwise introduce (Figure 4B).

**FIGURE 5:**
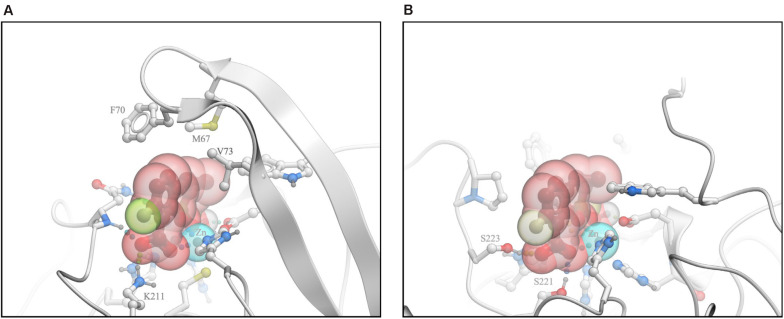
Analysis of interactions of QPX7728 with NDM-1 and L1 B-Lactamases. **(A)** The crystal structure of QPX7728/NDM-1 complex. **(B)** Model of the putative QPX7728/L1 complex. Modified from Lomovskaya et al. (2020b).

The crystal structure of QPX7728/NDM-1 complex (Hecker et al., 2020) revealed the key interactions that contribute to the high-affinity binding of the inhibitor to MBLs. Three distinct features can be discerned: (1) the ligand is coordinating both zinc ions at the core of the active site via one of the carboxylic acid’s oxygen atoms, the boronate ester oxygen, and its hydroxyl; (2) the ligand’s phenyl and cyclopropyl rings form extensive lipophilic contacts, largely with the sidechains in the loop L65:V73 that caps the active site; and (3) the ligand’s carboxylate forms a salt bridge and/or charge assisted hydrogen bonds with a positively charged sidechain/other hydrogen bond donors of the enzyme (Figure 5A).

As described in Lomovskaya et al. (2020b), we modeled the putative QPX7728/L1 complex by docking and attempted to identify the structural features that may preclude QPX7728 activity against β-lactamase L1. Docking simulation resulted in a bound pose of the ligand that retained coordination of the two metal ions in a manner closely resembling available experimental structures of complexes with other MBLs (Figure 5B). However, L1 structure lacks a positively charged sidechain analogous to K211 of NDM-1, therefore in the putative L1 complex QPX7728 did not form a salt bridge to the enzyme. Furthermore, QPX7728 hydrophobic interactions with L1 were limited due to the lack of the ‘capping’ loop in L1, where this loop is truncated to a short hairpin. Thus, it appears plausible that loss of lipophilic interactions with the ‘capping’ loop and the absence of a salt bridge to K211 (or similar residue), drastically reduce QPX7728 affinity to L1, in comparison to its affinity to most other MBLs. These observations suggest that significant modifications of QPX7728 chemical structure may be necessary to achieve potent inhibition of this sub-family of MBLs.

In conclusion, QPX7728 is a potent inhibitor of numerous β-lactamases from all molecular classes. Its β-lactamase inhibition profile is significantly enhanced compared to the recently approved BLIs. In addition to serine Class A, Class C and some Class D broad-spectrum β-lactamases and carbapenemases, the activity of QPX7728 includes serine Class D carbapenemases that are widely distributed in carbapenem-resistant *A. baumannii* (OXA-23/OXA-40) as well as metallo β-lactamases belonging to the B1 sub-class (NDM, VIM, IMP).

### QPX7728 Is Significantly Less Affected by General Intrinsic Resistance Mechanisms in Gram-Negative Bacteria

Gram-negative bacteria are inherently resistant to a variety of antibiotics (Nikaido, 2003; Li et al., 2015). This permanent multi-drug resistant state is due to a combined effect of the low permeability outer membrane and the activity of efflux transporters capable of recognizing and extruding multiple unrelated substrates, the so-called multi-drug resistance efflux pumps. B -lactams are one of the few classes of antibiotics that do have representatives with clinically useful activity against these multi-drug resistant gram-negative organisms; however, their activity can be decreased by permeability/efflux-mediated intrinsic mechanisms. B-lactamase inhibitors cannot overcome this type of resistance and thus their inhibitory potency might be decreased by these same intrinsic mechanisms (Shields et al., 2015; Nelson et al., 2017; Gomez-Simmonds et al., 2018; Wilson et al., 2019). Hence, the important objective of our BLI lead optimization program was to minimize the impact of these intrinsic resistance mechanisms on BLI potency.

#### Impact of Porin Mutations and Increased Efflux in *K. pneumoniae* on QPX7728 Potency

In Enterobacterales, major outer membrane porins OmpF/OmpK35 and OmpC/OmpK36 are important entry routes for β-lactams and β-lactamase inhibitors (Pages et al., 2008). Consequently, porin mutations affect the potency of many β-lactams and BL/BLI combinations (Lomovskaya et al., 2017). The pump which is the most relevant for β-lactam efflux is AcrAB (Pages et al., 2009). Using a set of KPC-producing porin/efflux mutants of *K. pneumoniae*, we demonstrated that combined effect of inactivation of the major porins OmpK35 and OmpK36 and increased efflux activity results in ca. 32-fold reduction of the potency of QPX7728 to completely inhibit KPC; the potency was reduced from 0.125 to 4 μg/ml (Lomovskaya et al., 2020a). This contrasts with a significantly stronger 256-fold reduction of the potency of vaborbactam by the same mutations (from 0.25 to 64 μg/ml) (Lomovskaya et al., 2017).

#### Impact of Efflux and OprD in *Pseudomonas aeruginosa* and Efflux in *Acinetobacter baumannii* on QPX7728 Potency

The outer membrane of *P. aeruginosa* lacks permanently open porins that are present in Enterobacterales (Chevalier et al., 2017) which significantly enhances its potential as permeability barrier. It also possesses multiple efflux pumps; working together these mechanisms provide the powerful protection against anti-pseudomonal toxins that include clinically useful antibiotics (Lister et al., 2009; El Zowalaty et al., 2015; Sommer et al., 2020). The MexAB-OprM efflux pump is particularly relevant for resistance to many β-lactam antibiotics (Srikumar et al., 1999), BLIs (Li et al., 1998), and BL/BLI combinations (Chalhoub et al., 2018; Castanheira et al., 2019). The basic amino acid porin OprD is used as a gate of entry of carbapenems (Ochs et al., 1999) and *oprD* mutants have an increased carbapenem resistance (Lister et al., 2009). Multiple multidrug resistance efflux pumps are described in *A. baumannii*: among them AdeABC and AdeIJK are particularly important for antibiotic resistance and general defense (Coyne et al., 2011).

In a panel of isogenic strains of *P. aeruginosa* that produced KPC (as a reporter) in the background of efflux/oprD mutations, we demonstrated that these mutations had a minimal effect on the BLI activity of QPX7728 (Lomovskaya et al., 2020a). No more than two-fold reduction in potency was observed when the *mexAB-oprM* efflux operon was overexpressed. For comparison, the potency of vaborbactam was reduced 8-fold or more due to overproduction of the MexAB-OprM or the MexEF-OprN efflux pumps, respectively (Lomovskaya et al., 2020a; Table 4). Inactivation of the carbapenem porin OprD did not have any effect on QPX7728 potency (Lomovskaya et al., 2020a).

**TABLE 4:** Evaluation of the impact of various efflux mutations on potency of QPX7728 (PV_50_) to enhance antibiotic activity.

Strain^1^	Genotype/Description	Biapenem^4^ MIC (μg/ml) in the presence of varied concentrations of BLIs (μg/ml)			MIC of the vector only strain (μg/ml)	QPX PV_50_^5^	VAB PV_50_^5^
		alone	w/QPX	w/VAB		(μg/ml)	(μg/ml)
PAM4224	*Wild-type*	64	0.06	4	0.25	1	8
PAM4365	*MexA::Tet*	64	0.125	0.25	0.25	1	0.5
PAM4126	*mexR* (MexAB-OprM)^2^	64	0.125	16	0.125	2	64
PAM4129	*nfxB* (MexCD-OprJ)	8	0.03	0.25	0.03	0.5	4
PAM4132	*mexT* (MexEF-OprM)^3^	128	0.25	32	0.5	1	>64
PAM4150	*mexZ* (MexXY-OprM)	64	0.25	4	0.25	1	8

*^1^KPC-2 was introduced into isogenic strains of P. aeruginosa containing various efflux mutations and used as a reporter for the QPX7728 BLI activity. ^2^Mutations in regulatory genes result in overproduction of specific efflux complexes that are shown in brackets. ^3^The production of a carbapenem porin OprD is reduced by the same mutation in mexT that leads to overproduction of the MexEF-OprN efflux pump. ^4^Biapenem was chosen as a reporter antibiotic because it’s activity against P. aeruginosa is not affected by efflux. The effect of increasing concentrations of QPX7728 on biapenem MIC against the strains producing KPC-2 in the background of various efflux mutations was determined in a standard checkerboard experiment. ^5^BLI potency was defined as PV_50_ (PV stands for potentiation value) which is a concentration of a BLI required to achieve 50% of antibiotic potentiation effect (conceptually equivalent to EC_50_). It is determined based on the concentration-response curve where antibiotic MICs are plotted vs. BLI concentrations. Data from Lomovskaya et al. (2020a).*

The experiments using isogenic panel of *Acinetobacter baumannii* mutants producing OXA-23 in the background of overexpression of efflux pumps also showed no effect of efflux on the potency of QPX7728 to inhibit β-lactamase (Lomovskaya et al., 2020a).

In conclusion, while mutations in major porins of Enterobacterales decrease the potency of QPX7728, it still maintains excellent inhibitory activity in strains with multiple porin mutations. Overexpression of efflux pumps in various target organisms (Enterobacterales, Pseudomonas, Acinetobacter) does not reduce the antibiotic potentiation activity of QPX7728.

### QPX7728 in Combination With Various β-Lactams Against Clinical Isolates of Target Pathogens

Several studies were performed to determine the potency of QPX7728 in combination with various β-lactams against large panels of clinical isolates of target pathogens. We used a newly developed metric called target potentiation concentration or TPC [described in detail in Nelson et al. (2020a)] to separately determine the whole-cell BLI potency of QPX7728. TPC is the concentration of QPX7728 that is required to reduce antibiotic MIC for a given strain to or below the existing susceptibility breakpoint of the partner antibiotic tested. Conceptually, it is the MIC of the BLI determined in the presence of a partner antibiotic present at a concentration that corresponds to its susceptibility breakpoint. By assessing the distribution of TPCs in the population of strains, one can determine TPC_90_, or the concentration of QPX7728 that shifts 90% of the tested isolates at or below susceptibility breakpoint for the partner antibiotic. This metric is extremely useful when one wants to compare the potency of a potentiating agent in combination with different antibiotics against different panels of target organisms and to evaluate the impact of various genotypes and phenotypes on its potency.

In addition, TPC_90_ is an important metric that will be used to link *in vitro* BLI potency to exposures required for BLI activity *in vivo*. Once safe and efficacious BLI exposures established based on TPC_90_ and various PK-PD studies are achieved, TPC_90_ will be also important to select concentration of QPX7728 for *in vitro* susceptibility testing.

### QPX7728 Restores the Potency of Various β-Lactam Antibiotics Against Enterobacterales With Diverse Acquired and Intrinsic Mechanisms of β-Lactam Resistance

**FIGURE 6:**
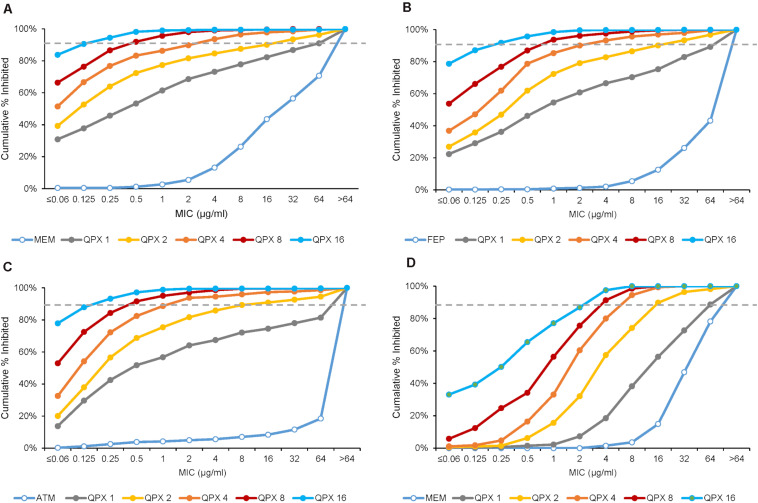
MIC distributions of various antibiotics with increasing concentrations of QPX7728 against ESBL and carbapenemase-producing Enterobacterales **(A–C)** and carbapenem-resistant *Acinetobacter baumannii* **(D)**. **(A)** Meropenem alone and with QPX7728 tested against the panel of 598 CRE isolates that contained 285 KPC, 39 OXA-48, 50 non-carbapenemase-producing CRE and 224 MBL-producing strains (149 NDM, 51 VIM, 19 IMP). **(B)** Cefepime or aztreonam **(C)** alone and in combination with QPX7728 tested against the panel of 756 strains of *Enterobacterales* with characterized β-lactamases; in addition to class A ESBLs and various class C enzymes it contained 302 KPC (9 in combination with MBLs), 70 OXA-48-like and 224 MBLs. **(D)** Meropenem alone and with QPX7728 tested against the panel of 275 carbapenem-resistant strains of *A. baumannii*. Figures are constructed based on data in Nelson et al. (2020a; 2020b).

In the study by Nelson et al. (2020a), increasing concentrations of QPX7728 (1–16 μg/ml) were shown to significantly enhance the potency of meropenem against a panel of 598 molecularly characterized carbapenem-resistant strains of Enterobacterales (CRE), consisting of serine and metallo β-lactamases producers as well as non-carbapenemase producing CREs (Figure 6A). This study determined the TPC_90_ of QPX7728 for this panel of strains using meropenem susceptibility breakpoint of 8 μg/ml [meropenem PK-PD breakpoint at a dose of 2g administered every 8 h by 3-h infusion (Kuti et al., 2003; Lee et al., 2010)]. The TPC_90_ of QPX7728 for studied CRE isolates was 4 μg/ml.

**FIGURE 7:**
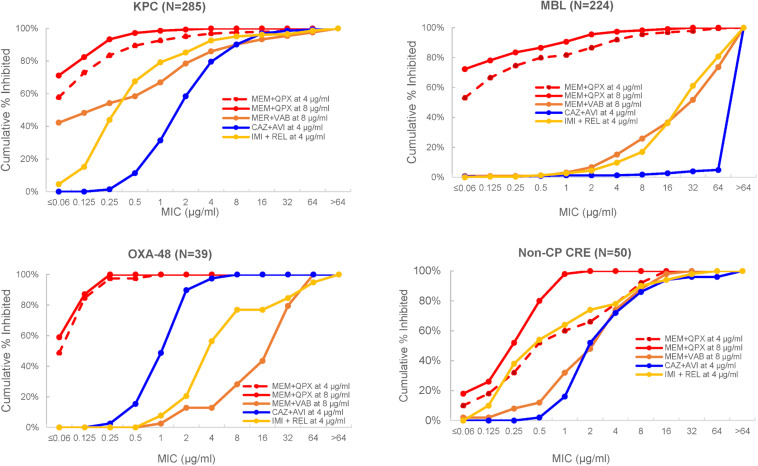
MIC distributions of various β-lactam/BLI combinations against carbapenem-resistant *Enterobacterales* according to the type of CRE. MEM, meropenem; CAZ, ceftazidime; IMI, imipenem; AVI, avibactam; QPX, QPX7728; REL, relebactam; Non-CP CRE, non-carbapenemase producing CRE. Adapted with modifications from Nelson et al. (2020a).

Importantly, the potency of meropenem was increased by QPX7728 against all groups of carbapenemase producing CREs, consisting of KPC-, OXA-48-like- and MBL-producing strains, as well as against non-carbapenemse-producing CRE strains. MIC_90_ of meropenem was 1 μg/mL or less when QPX7728 was tested at 8 μg/ml; at this concentration of QPX7728 (as well as at 4 μg/ml) meropenem/QPX7728 was the most potent BL/BLI combination tested against all groups of CRE (Figure 7).

As expected from the studies of laboratory mutants (Lomovskaya et al., 2020a), porin mutations reduced the potency of meropenem/QPX7728. However, when meropenem was combined with QPX7728 at 4 or 8 μg/ml, more than 90% of both MBL-negative and MBL-positive CRE with various defects in OmpK36/OmpC were susceptible using a meropenem breakpoint of 8 μg/ml (Table 5); thus, the QPX7728 TPC_90_ values were still in the 4–8 μg/ml range even for the subsets of strains that had a combination of carbapenemase production and non-β-lactamase-mediated resistance mechanisms.

**TABLE 5:** *In vitro* activity (MIC_50_/MIC_90_ and % inhibited) of meropenem alone and combined with QPX7728 at 4 and 8 μg/ml and comparator BLI combination agents against carbapenem resistant strains of *Enterobacterales* according to the functional status of OmpK35/OmpF and OmpK36/OmpC and carbapenemase present.

	Meropenem-QPX7728 at 4 μg/ml	Meropenem-QPX7728 at 8 μg/ml	Meropnem-vaborbactam at 8 μg/ml	Ceftazidime-avibactamat 4 μg/ml	Imipenem-relebactam at 4 μg/ml
***K. pneumonia* KPC (*N* = 230)**					
OmpK36-defective (*N* = 135)	0.25/2	0.125/0.5	2/32	4/16	0.5/4
% Inhibited	94.8%	100.0%	72.6%	88.1%	70.4%
OmpK36-functional (*N* = 95)	≤0.06/0.125	≤0.06/≤0.06	≤0.06/0.25	2/8	0.25/1
% Inhibited*	100.0%	100.0%	97.9%	91.6%	94.7%
**MBL (*N* = 177)**					
OmpK36-defective (*N* = 42)	4/32	1/4	>64/>64	>64/>64	>64/>64
% Inhibited	81.0%	92.9%	0.0%	0.0%	0.0%
OmpK36-functional (*N* = 135)	≤0.06/0.5	≤0.06/0.125	32 > 64	>64/>64	32>64
% Inhibited	99.3%	99.3%	20.0%	2.2%	3.7%

*: % inhibited at the following concentrations: meropenem-QPX7728: ≤ 8/4 μg/ml and ≤ 8/8 (PK-PD susceptible breakpoint); meropenem-vaborbactam: ≤ 4/8 μg/ml (FDA susceptible breakpoint); ceftazidime-avibactam: ≤ 8/4 μg/ml (FDA susceptible breakpoint); imipenem-relebactam: ≤ 1/4 μg/ml (FDA susceptible breakpoint). Frame-shift and nonsense mutations, insertions of IS elements in the structural part or in a promoter sequence, small amino-acid insertions constricting the channel were identified as mutations resulting in a defective protein. In the absence of these mutations, OmpK36 was assumed to be functional. Data from Nelson et al. (2020a).

We also assessed enhancement of the potency of cefepime and aztreonam by QPX7728 (at concentrations from 1 to 16 μg/ml) against the panel of 756 of ESBL and CRE producing strains of Enterobacterales with the objective to determine the TPC_90_ of QPX7728 in combination with these antibiotics.

QPX7728 significantly increased potency of both antibiotics against this challenge panel: MIC_90_ for both antibiotics was decreased from >64 to 2 μg/ml and 0.25 μg/ml with QPX7728 at 4 and 8 μg/ml, respectively (Figures 6B,C). The QPX7728 TPC_90_, determined at 8 μg/ml of both antibiotics (FDA susceptible breakpoint), was 4 μg/ml in combination with either cefepime or aztreonam.

Overall, combinations of QPX7728 with multiple antibiotics were highly potent against the majority of strains, including those which had various carbapenemases produced in the background of mutations resulting in decreased permeability. The potency of QPX7728 as measured by TPC_90_ was the same, irrespective of the antibiotic combined with QPX7728.

### QPX7728 in Combination With Several β-Lactams Has Excellent Potency Against KPC Mutants That Are Resistant to Ceftazidime-Avibactam

Mutations in KPC genes that confer resistance to ceftazidime-avibactam were first selected in laboratory conditions (Livermore et al., 2015), but soon were identified in isolates recovered from patients treated with this combination (Shields et al., 2017). Based on several studied cases, this resistance appears to be due to both an increase in the efficiency of ceftazidime hydrolysis by KPC and reduced affinity of the avibactam/KPC interaction (Barnes et al., 2017; Tsivkovski and Lomovskaya, 2020). Using a set of single-step ceftazidime-avibactam resistant mutants with various mutations in the *bla*_*KPC*_ selected from a diverse KPC-producing strains of *Klebsiella pneumoniae*, we demonstrated that these mutations had minimal effect on the MIC of QPX7728 in combination with ceftazidime, cefepime or meropenem (Nelson et al., 2019). Depending on the strain or specific mutation, ceftazidime-avibactam MIC values were raised from 4 to >64-fold, resulting in MIC values ranging from 16 to >128 μg/ml (Table 6). The majority of mutants had no increase in ceftazidime-QPX7728, meropenem-QPX7728 or cefepime-QPX7728 MIC values. Meropenem-QPX7728 and cefepime-QPX7728 MIC values of the mutants ranged from ≤ 0.125 to 0.25 μg/ml and 0.25 to 1 μg/ml, respectively. The highest observed ceftazidime-QPX7728 MIC for the mutants was 4 μg/ml (due to KPC-2::D179Y (KPC-31) mutation selected from KP1099, ceftazidime-avibactam MIC > 128 μg/ml).

**TABLE 6:** The effect of KPC mutations selected with ceftazidime-avibactam on potency of QPX7728 in combination with ceftazidime, meropenem, and cefepime.

Strain	B-lactamases	CAZ	CAZ+AVI	CAZ+QPX	MEM	MEM+QPX	FEP	FEP+QPX
**KP1074**	KPC-3, SHV-11, TEM-1/GD	>128	4	0.5	128	≤0.125	>128	0.5
KPM3200	KPC-3::duplication of EAVI (aa#278)	>128	64	0.5	64	≤0.125	>128	0.5
KPM3205	KPC-3 ::duplication of KKDY (aa#273)	>128	128	1	16	≤0.125	128	0.5
**KP1099**	KPC-2, SHV-11, SHV-12, CTX-M-14/GD	>128	2	1	128	≤0.125	>128	0.25
KPM3210	KPC-2::D179N	>128	32	0.5	4	≤0.125	128	≤0.125
KPM3213	KPC-2::D179Y (KPC-31)	>128	>128	4	4	≤0.125	>128	0.25
KPM3214	KPC-2::D179A	>128	128	2	2	≤0.125	128	0.5
KPM3212	deletion of ELNSAI at aa#497	>128	64	2	1	≤0.125	128	0.5
**KP1252**	KPC-3, TEM-1, SHV-11/FL	>128	8	1	64	≤0.125	>128	0.5
KPM3512	KPC-3:: P174L	>128	32	2	8	≤0.125	16	≤0.125
KPM3511	KPC-3::P174Q	>128	64	1	4	0.25	64	0.5
**KP1317**	KPC-3, TEM-1, SHV-11/GD	>128	2	1	>128	≤0.125	>128	0.5
KPM3517	KPC-3::D179Y (KPC-31)	>128	64	1	128	≤0.125	>128	0.5
KPM3520	Both WT and mutant KPC-3::duplication of 8aa (aa#264–271)	>128	64	1	128	0.25	>128	0.5
KPM3523	Both WT and mutant KPC-3:: deletion of EL (aa#241–242)	>128	32	1	128	≤0.125	128	1
**KP1500**	KPC-3, TEM-1, SHV-11, CTX-M-15 OXA-1 OXA-9/IS at −48	>128	4	0.5	>128	≤0.125	>128	0.5
KPM3546	KPC-3::R164H	>128	32	1	128	0.25	>128	0.5
**KP1102**	KPC-3, TEM-1, SHV-11/GD	>128	4	1	>128	≤0.125	>128	4
KPM3493	KPC-3::duplication of EL at aa#164 (KPC-25)	>128	16	2	1	≤0.125	32	1
KPM3494	KPC-3::R167S	>128	32	1	32	≤0.125	128	1
KPM3495	KPC-3::A175V	>128	32	1	32	≤0.125	>128	0.5
**KP1246**	KPC-3, SHV-12, SHV-11/FL	>128	8	0.25	128	≤0.125	128	0.25
KPM3191	KPC-3:: DDK duplication at aa#831 (KPC-29)	>128	64	≤0.125	4	≤0.125	64	0.25
KPM3192	KPC-3:: D179Y (KPC-31)	>128	128	0.25	0.5	≤0.125	32	0.25

*Avibactam and QPX7728 tested at 4 μg/ml. bla_KPC_ was detected in two different locations in the strain KP1317. Parent strains are in bold font. Data from Nelson et al. (2019).*

Additional studies are underway to directly assess the interaction of QPX7728 with various KPC variants that confer resistance to ceftazidime-avibactam. However, it appears that QPX7728 combined with multiple antibiotics is capable to overcome this resistance.

### QPX7728 Restores Potency of Meropenem Against Carbapenem-Resistant *Acinetobacter baumannii* (CRAB)

A collection of 275 clinical isolates of highly carbapenem resistant *Acinetobacter baumannii* (MIC_50_/MIC_90_ for meropenem of 64/>64 μg/ml) was used to assess the enhancing effect of QPX7728 on meropenem potency (Nelson et al., 2020b). Combining meropenem with increasing concentrations of QPX7728 (1–16 μg/ml) resulted in a >8–16-fold increase in meropenem potency: MIC_90_ of meropenem decreased to 8 and 4 μg/ml by addition of QPX7728 at 4 and 8 μg/ml, respectively (Figure 6D).

Numerous studies demonstrated that mutations in penicillin binding proteins, inactivation of porin genes and increased efflux are the most relevant non-β-lactamase-mediated mechanisms associated with reduced activity of meropenem against *A. baumannii* (Vila et al., 2007; Hawkey et al., 2018). We have found that the same mechanisms also increased the MIC values of meropenem/QPX7728 (Nelson et al., 2020b). Mutations in PBP3 that were mapped to the substrate-binding site appear to have the strongest impact on its potency increasing 4–8-fold the MIC_90_ of meropenem/QPX7728. Nevertheless, MIC_90_ for the strains that produced carbapenemases in the background of PBP3 mutations was still below the PK-PD breakpoint for meropenem (8 μg/ml). We also noted that when PBP3 mutations were absent, meropenem-QPX7728 MICs values were less than or equal to 8 μg/ml and in the absence of both PBP3 and efflux regulatory mutations, meropenem-QPX7728 MIC values were less than or equal to 4 μg/ml. We believe that the systematic evaluation of resistance mechanisms and their impact on MIC will facilitate the future development of molecular tools that can be used to predict susceptibility and resistance to QPX7728 combinations.

The study described above (Nelson et al., 2020b) also allowed the determination of the TPC_90_ of QPX7728 in combination with meropenem: it was less than or equal to 8 μg/ml for the majority of the subsets of strains stratified according to the presence of various intrinsic mechanisms, similar to what was observed in carbapenem-resistant Enterobacterales.

The plasma QPX7728 exposure associated with 8 μg/ml was found to be efficacious in animal models of infection (Sabet et al., 2020), and is being targeted in human dosage regimens during ongoing clinical development^1^. Thus, subsequent microbiological evaluation of QPX7728 in combination with IV antibiotics was performed using QPX7728 at a fixed concentration of 8 μg/ml. We expect this concentration of QPX7728 will be adopted for future *in vitro* susceptibility testing using various manual, semiautomatic and automatic systems.

### QPX7728 Enhances Potency of Multiple β-Lactam Antibiotics Against *Pseudomonas aeruginosa*

The study described by Lomovskaya et al. (2021) investigated the impact of QPX7728 on potency of meropenem, cefepime and ceftolozane against two panels of *P. aeruginosa*. The first panel consisted of 500 isolates that were selected to mirror MIC distributions of β-lactams and BL/BLI combinations observed in recent global surveillance studies. QPX7728 shifted > 90% of these isolates below susceptibility breakpoint of 8 μg/ml for meropenem and cefepime or 4 μg/ml for ceftolozane (Table 7). In agreement with surveillance reports (Sader et al., 2020), ceftolozane-tazobactam and ceftazidime-avibactam were also highly potent, inhibiting > 90% of these isolates at their susceptibility breakpoints.

**TABLE 7:** *In vitro* potency of various antibiotics alone and in combination with QPX7728 at 8 μg/ml and comparator agents against representative and challenge panels of clinical isolates of *Pseudomonas aeruginosa*.

The panel reflecting current MIC distribution for β -lactams and BL/BLI combinations (*N* = 500)									

	MEM	QPX/MEM	TOL	TOL-TAZ	QPX/TOL	FEP	QPX/FEP	CAZ-AVI	PIP-TAZ
MIC_50_ (μg/ml)	0.5	0.25	0.5	0.5	0.5	4	2	2	8
MIC_90_ (μg/ml)	16	8	4	4	1	32	8	8	128
% Inhibited^∗^	84.8%	91.6%	90.4%	91.8%	97.6%	74.4%	91.2%	92.2%	71.6%

The challenge panel (*N* = 290)															

All (*N* = 290)		MEM	QPX/MEM	ATM	QPX/ATM	PIP	QPX/PIP	TOL	QPX/TOL	FEP	QPX/FEP	PIP-TAZ	TOL-TAZ	CAZ-AVI	IMP-REL
	MIC_50_	16	8	32	16	>64	16	4	1	32	8	>64	4	8	4
	MIC_90_	>64	64	>64	64	>64	64	>64	64	64	64	>64	>64	>64	64
No MBL (*N* = 229)	% Inhibited*	33.1	60.3	19.0	45.9	16.2	70.3	51.4	78.6	20.0	70.3	17.2	52.1	50.7	46.9
	MIC_50_	16	8	32	16	>64	16	2	1	32	8	>64	2	8	2
	MIC_90_	64	32	>64	32	>64	64	>64	4	64	16	>64	>64	64	16
MBL (*N* = 61)	% Inhibited*	41.9	68.1	17.9	46.7	19.7	69.9	64.2	92.1	23.6	79.5	20.1	65.5	62.9	59.0
	MIC_50_	>64	32	32	16	>64	16	>64	64	64	32	>64	>64	>64	>64
	MIC_90_	>64	>64	>64	64	>64	64	>64	>64	64	64	>64	>64	>64	>64
	% Inhibited*	0.0	31.1	23.0	42.6	3.3	72.1	3.3	27.9	6.6	36.1	6.6	1.6	4.9	1.6

MEM, meropenem; QPX, QPX7728; TOL, ceftolozane; TAZ, tazobactam; FEP, cefepime; CAZ-AVI, ceftazidime-avibactam; PIP-TAZ, piperacillin-tazobactam; tazobactam and avibactam were tested at fixed 4 μg/ml. *: % inhibited at the following concentrations: meropenem: ≤ 8 μg/ml; QPX7728/meropenem: ≤ 8 μg/ml with QPX7728 at 8 μg/ml; ceftolozane: ≤ 4 μg/ml; QPX7728/ceftolozane: ≤ 4 μg/ml with QPX7728 at 8 μg/ml; ceftolozane-tazobactam: ≤ 4/4 μg/ml; cefepime: ≤ 8 μg/ml; QPX7728/cefepime: ≤ 8 μg/ml with QPX7728 at 8 μg/ml; ceftazidime-avibactam: ≤ 8/4 μg/ml; piperacillin-tazobactam: ≤ 16 μg/ml. Data from Lomovskaya et al. (2021).

The enhancement of potency of multiple β-lactams by QPX7728 was also observed against the second panel of strains (N = 290) consisting of meropenem or ceftolozane-tazobactam non-susceptible or ceftazidime-avibactam resistant isolates. QPX7728 in combination with meropenem, ceftolozane, cefepime and piperacillin inhibited a higher percentage strains at the susceptibility breakpoint for the partner β-lactam alone compared to clinically used BL/BLI combinations. In general, the highest activity was observed for QPX7728-ceftolozane, with 78.6% of isolates inhibited at 4 μg/ml of ceftolozane. 70.3% of isolates were shifted by QPX7728 at or below 8 or 16 μg/ml for cefepime and piperacillin, respectively, and 60.3% of isolates were inhibited by QPX7728-meropenem at MIC of ≤ 8 μg/ml (Table 7).

The result from this study is that the activity of QPX7728/β-lactam combinations will be determined by multiple factors. First, the liability of a specific β-lactam to hydrolysis of a particular β-lactamase, i.e., if the β-lactamase is a cephaloporinase vs. carbapenemase vs. penicillinase. Second, the inhibitory potency of QPX7728 for a particular β-lactamase. Finally, the impact of non- β-lactamase mediated β-lactam resistance mechanisms such as increased efflux by MexAB-OprM or inactivation of OprD on both the inhibitor and β-lactam will also impact activity (Lomovskaya et al., 2021).

Data obtained in biochemical experiments indicate that QPX7728 inhibits serine β-lactamases with higher potency than metallo enzymes, consistent with higher activity of QPX7728-meropenem, QPX7728-ceftolozane and QPX7728-cefepime against MBL-negative than MBL-positive strains. Data from MIC experiments using cloned β-lactamases indicate that potentiation with QPX7728 is more effective with antibiotics that are less susceptible to β-lactamase-mediated hydrolysis. This is consistent with the higher potency of QPX7728-piperacillin (piperacillin appears to be less affected by MBL-mediated hydrolysis compared to ceftolozane, cefepime and meropenem) combination against the MBL-positive strains compared to other QPX7728 combinations. As expected, the potency of QPX-aztreonam does not depend on the presence or absence of MBLs as aztreonam is not hydrolyzed by these enzymes.

Studies with isogenic strains demonstrated that various combinations of non- β-lactamase-mediated intrinsic mechanisms such as increased efflux by MexAB-OprM or inactivation of a carbapenem-specific porin OprD may raise the MICs of some β-lactams to or above their susceptibility breakpoints (Lomovskaya et al., 2021). The combination of QPX7728 plus ceftolozane is the most potent combination against MBL-negative strains of *Pseudomonas aeruginosa* since neither drug is a substrate of efflux nor is affected by inactivation of OprD. In contrast, meropenem MICs can be raised to or above the PK-PD breakpoint of 8 μg/ml when overexpression of the MexAB-OprM efflux pump is combined with inactivation of OprD. These observations are important for the potential future use of β-lactamase inhibitors; different β-lactams can be chosen as companion antibiotics for QPX7728 to achieve the optimal therapeutic effect. Co-administration of QPX7728 with a specific β-lactam will depend on resistance mechanisms identified in the specific pathogen. Undoubtedly, timely selection of the best companion β-lactam at the individual strain/patient level requires availability of efficient susceptibility testing methods and can be facilitated by the development of molecular tools based on deep understanding of the impact of numerous resistance mechanisms on antibiotics potency. This strategy might give physicians more options when managing infections due to highly resistant organisms, particularly in patients with limited choices.

### QPX7728 Enhances the Potency of Oral β-Lactam Antibiotics Against Enterobacterales Producing ESBLs or Carbapenemases

QPX7728 has 43–53% oral bioavailability in rats at doses of 30–100 mg/kg (Hecker et al., 2020) and an oral formulation is being developed for oral administration. Using a large panel of ESBL and carbapenemase-producing strains of Enterobacterales we demonstrated (Rubio-Aparicio et al., 2019) that QPX7728 significantly enhanced the potency of multiple β-lactams against these strains (Table 8).

**TABLE 8:** *In vitro* activity of QPX7728 in combination with oral β-lactams against *Enterobacterales* producing ESBLs and carbapenemases.

Organism	MIC_50/90_ (μg/ml)					
	Cefpodoxime	Cefpodoxime-QPX7728	Ceftibuten	Ceftibuten-QPX7728	Tebipenem	Tebipenem-QPX7728
All Enterobacterales (*N* = 972)	>64/>64	1/16	32/>64	≤0.06/4	0.25/>64	≤0.06/1
ESBLs, no CRE (*N* = 371)	>64/>64	0.5/4	8/>64	≤0.06/1	≤0.06/0.5	≤0.06/0.125
CRE KPC (*N* = 292)	>64/>64	2/8	16/>64	0.125/0.5	64/>64	≤0.06/2
CRE OXA-48 (*N* = 47)	>64/>64	1/8	64/>64	0.125/1	32/64	0.25/0.5
CRE MBL (*N* = 226)	>64/>64	>64/>64	>64/>64	2/>64	32/>64	0.125/32
Non-CP CRE	>64/>64	>64/>64	>64/>64	2/>64	8/16	1/4

*Non-CP CRE, no carbapenemasee-producing CRE; QPX7728 tested at 4 μg/ml. Data from Rubio-Aparicio et al. (2019).*

## Conclusion

In conclusion, QPX7728 is a novel ultra-broad-spectrum β-lactamase inhibitor with the broadest spectrum of inhibition reported up to date in a single BLI molecule. The ability to inhibit metallo enzymes did not come at the expense of reduced spectrum or potency against serine β-lactamases. On the contrary, the spectrum of inhibition of serine enzymes by QPX7728 was also expanded compared to that of the clinically available BLIs – it has a potent activity against serine enzymes that historically have been difficult to inhibit such as class D carbapenemases from *A. baumannii* (*e.g.*, OXA-23). Importantly it is minimally affected by general intrinsic resistance mechanisms such as efflux and porin mutations. QPX7728 combinations with several IV β-lactam antibiotics showed high potency against strains of Enterobacterales, *Acinetobacter baumannii* and *Pseudomonas aeruginosa* that are resistant to other recently approved IV β-lactam-BLI combinations, e.g., ceftazidime-avibactam, ceftolozane-tazobactam, meropenem-vaborbactam and imipenem-relebactam. Due to its broad β-lactamase inhibition spectrum and the ability to potentiate multiple antibiotics against multiple target pathogens, QPX7728 is suited perfectly for a stand-alone BLI strategy, where it can be combined with multiple different β-lactams, with the goal of providing more optionality for both treatment and stewardship efforts. QPX7728 can be delivered orally, and in combination with oral β-lactams has promising activity against ESBL and carbapenemase-producing strains of Enterobacterales ensuring that ultra-broad-spectrum β-lactamase inhibition spectrum and other features of QPX7728 could be applied to both IV and oral QPX7728-based products. Clinical development of QPX7728 has been initiated.

## Author Contributions

OL wrote the manuscript with critical feedback from all authors. MT, JL, and MD contributed to the final version of the manuscript. All authors are members of the team that is developing QPX7728 and contributed to the results described in this overview.

## Conflict of Interest

OL, RT, DS, RR, SH, DG, JL, and MD are employees and shareholders of Qpex Biopharma that is developing QPX7728. MT is an employee of Molsoft, LLC. The remaining author declares that the research was conducted in the absence of any commercial or financial relationships that could be construed as a potential conflict of interest.

## Footnotes

1
https://clinicaltrials.gov/ct2/show/NCT04380207
